# Linking dysbiosis to precancerous stomach through inflammation: Deeper than and beyond imaging

**DOI:** 10.3389/fimmu.2023.1134785

**Published:** 2023-03-31

**Authors:** Catarina Lopes, Tatiana C. Almeida, Pedro Pimentel-Nunes, Mário Dinis-Ribeiro, Carina Pereira

**Affiliations:** ^1^ Precancerous Lesions and Early Cancer Management Group, Research Center of IPO Porto (CI‐IPOP)/Rise@CI‐IPOP (Health Research Group), Portuguese Institute of Oncology of Porto (IPO Porto)/Porto Comprehensive Cancer Center (Porto.CCC), Porto, Portugal; ^2^ CINTESIS – Center for Health Technology and Services Research, University of Porto, Porto, Portugal; ^3^ ICBAS-UP – Institute of Biomedical Sciences Abel Salazar, University of Porto, Porto, Portugal; ^4^ Department of Surgery and Physiology, Faculty of Medicine, University of Porto (FMUP), Porto, Portugal; ^5^ Department of Gastroenterology, Unilabs, Porto, Portugal; ^6^ Department of Gastroenterology, Portuguese Institute of Oncology of Porto, Porto, Portugal

**Keywords:** atrophic gastritis, biomarkers, inflammation, microbiota, functional endoscopy

## Abstract

Upper gastrointestinal endoscopy is considered the gold standard for gastric lesions detection and surveillance, but it is still associated with a non-negligible rate of missing conditions. In the Era of Personalized Medicine, biomarkers could be the key to overcome missed lesions or to better predict recurrence, pushing the frontier of endoscopy to functional endoscopy. In the last decade, microbiota in gastric cancer has been extensively explored, with gastric carcinogenesis being associated with progressive dysbiosis. *Helicobacter pylori* infection has been considered the main causative agent of gastritis due to its interference in disrupting the acidic environment of the stomach through inflammatory mediators. Thus, does inflammation bridge the gap between gastric dysbiosis and the gastric carcinogenesis cascade and could the microbiota-inflammation axis-derived biomarkers be the answer to the unmet challenge of functional upper endoscopy? To address this question, in this review, the available evidence on the role of gastric dysbiosis and chronic inflammation in precancerous conditions of the stomach is summarized, particularly targeting the nuclear factor-κB (NF-κB), toll-like receptors (TLRs) and cyclooxygenase-2 (COX-2) pathways. Additionally, the potential of liquid biopsies as a non-invasive source and the clinical utility of studied biomarkers is also explored. Overall, and although most studies offer a mechanistic perspective linking a strong proinflammatory Th1 cell response associated with, but not limited to, chronic infection with *Helicobacter pylori*, promising data recently published highlights not only the diagnostic value of microbial biomarkers but also the potential of gastric juice as a liquid biopsy pushing forward the concept of functional endoscopy and personalized care in gastric cancer early diagnosis and surveillance.

## 1 An overview: Beyond and before imaging

Gastric cancer (GC) remains one of the deadliest cancers in the world. It became the 6^th^ most diagnosed cancer type in 2020, with approximately 1.09 million new cases worldwide, and 769 thousand cancer-related deaths, data revealed by the International Agency for Research on Cancer (IARC) (1). Although GC rates have decreased over the last few decades, GC continues to be a major concern for public health, with an expected increase in the burden due to its prevalence and high mortality-to-incidence ratio associated with its detection at advanced stages (2).

GC develops through a series of steps caused by the exposure and interaction of several factors, often referred to as the “Correa Cascade” (3). Most of these factors have a cumulative effect on the gastric mucosal microenvironment, triggering degenerative changes, starting with chronic gastritis, most often caused by *Helicobacter pylori (H. pylori)* infection, and progressing *via* atrophy (AG), intestinal metaplasia (IM), dysplasia, and finally, the development of cancer (4). Genes involved in cell adhesion, signal transduction, differentiation, development, and DNA repair are all susceptible to being mutated by carcinogens throughout the carcinogenesis process (5). Somatic gene mutations or single nucleotide polymorphisms (SNPs) and epigenetic modifications involving DNA methylation are just a few examples of the wide range of important genetic effects that contribute to the molecular pathogenesis (6). These alterations are theorized to drive the progression of cells from stage to stage, culminating in the development of cancer (6).

**Figure 1:**
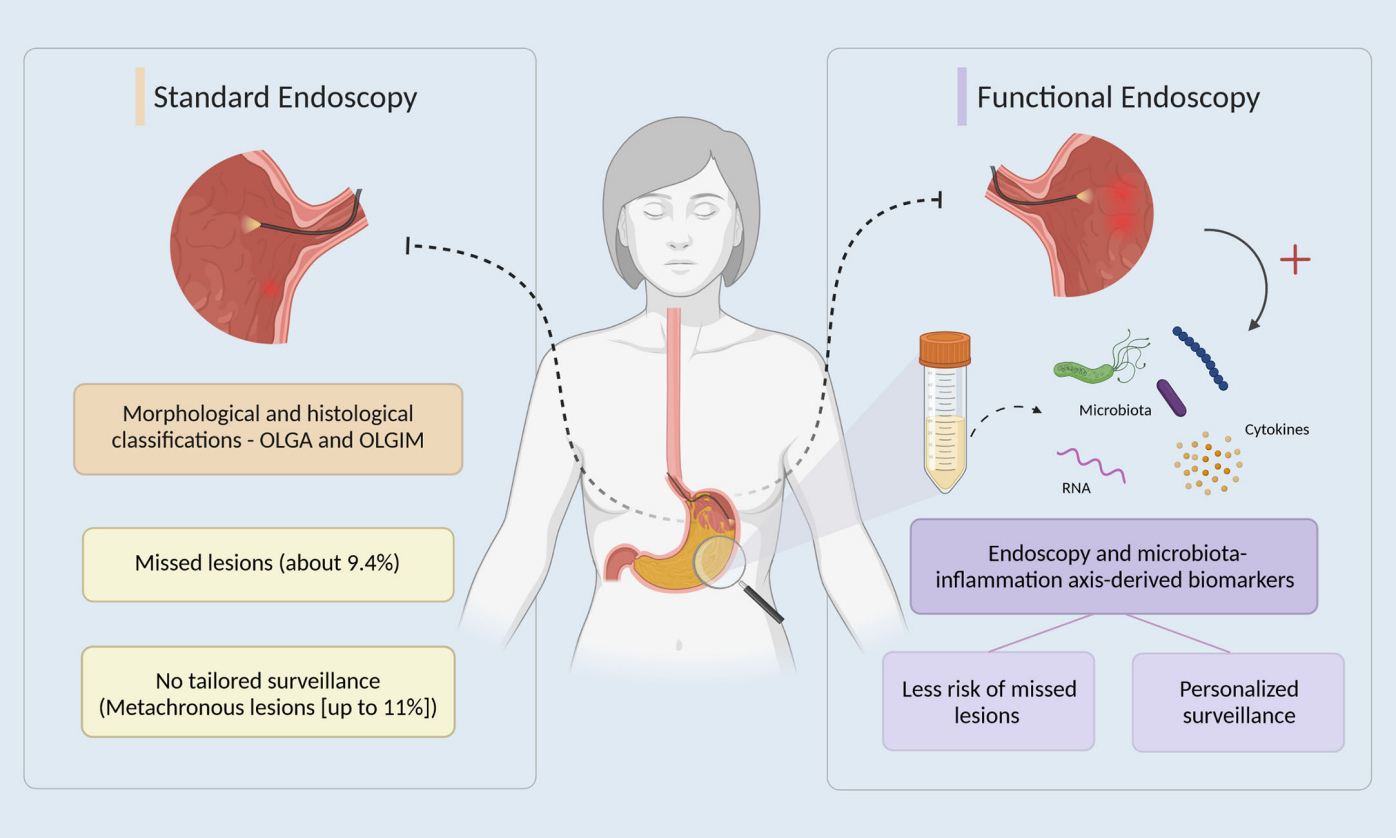
The potential of functional endoscopy in gastric cancer early detection and management. In standard endoscopy (left side), after an upper gastrointestinal endoscopy, patients are stratified using OLGA and OLGIM classifications, which rank the severity and topography of atrophic and IM alterations in gastritis (13). However, endoscopy alone is associated with a non-negligible rate of missed conditions and a significant risk of metachronous lesions development (15, 16). On the right side, the promise of functional endoscopy by also integrating, for example, dysbiosis-driven inflammatory biomarkers derived from liquid biopsies, namely gastric juice, towards the individualization of care by targeting a reduction of missed lesions and personalization of patients’ management in gastric cancer early detection.

The detection of early stages in this multistep process, namely AG and IM, is of utmost importance, since it allows for less invasive and more effective treatments, leading to increased survival (76% in Korea, where 90% of cancers are diagnosed early vs 25% in Europe) and quality of life of patients, and reduces the economic burden associated with the treatment of more advanced stages (7–10). Several strategies are available for the early detection of GC, from non-invasive detection of circulating biomarkers to upper digestive endoscopy (10, 11). The latter is considered the gold standard, although proven not cost-effective for screening outside high-risk regions (12). Moreover, patients at higher risk of developing early GC can be stratified using endoscopic and histologic classifications like the operative link on gastritis assessment (OLGA), and the operative link on gastric intestinal metaplasia assessment (OLGIM), which rank the severity and topography of atrophic and IM alterations in gastritis, respectively, from stages 0 to IV, with stage III or IV considered to be high risk for GC development (13). In fact, in Europe and according to published guidelines the endoscopic surveillance of patients diagnosed with stomach precancerous conditions/lesions is endorsed as a strategy to detect GC when minimally invasive treatment is possible (14). However, the upper gastrointestinal (GI) endoscopy is still associated with a non-negligible rate of missed conditions (about 9.4 %), as reported in the systematic review and meta-analysis by Pimenta-Melo et al. (15), and the minimally invasive removal of early lesions is associated with a significant risk of metachronous lesions development (16). In the Era of Personalized Medicine, should other biomarkers besides age, sex, family history and morphology/histology be added to the equation? And when should we do it? When first performing the endoscopy to overcome missed lesions? Or during surveillance to better predict recurrence/secondary lesions? Could the microbiota-inflammation axis-derived biomarkers challenge the purely histological and imaging nature of current approaches towards the concept of functional endoscopy and individualization of care (**Figure 1**)?

To tackle these questions, we reviewed and will, in the next sections, summarize the available evidence on the role of gastric dysbiosis and chronic inflammation, particularly targeting the nuclear factor-κB (NF-κB), toll-like receptors (TLRs) and cyclooxygenase-2 (COX-2) pathways, in the early phases of gastric carcinogenesis. The potential of liquid biopsies as a non-invasive source, as well as the clinical utility of studied biomarkers, will be explored in the last sections.

## 2 Gastric dysbiosis as a main player in early gastric carcinogenesis

The predominant role of *H. pylori* in GC development has triggered an increasing interest in the study of gastric dysbiosis in last decade (17). In fact, gastric microbiota composition is highly variable between individuals (18). However, current research has found five main phyla in the normal stomach, including *Firmicutes*, *Bacteroidites*, *Actinobacteria*, *Fusobacteria*, and *Proteobacteria*, with *Prevotella*, *Streptococcus*, *Veillonella*, *Rothia*, and *Haemophilus* as the most common taxa (19–23). Human health depends on maintaining a balanced microbiome (18). Dysbiosis, characterized by alterations in microbiome content, has been linked to a broad set of disorders, including cancer (24). Studies that examined the gastric microbiome in the context of GC identified several bacterial communities characterized by different species (25, 26). Although several studies have documented stomach microbiota in GC patients, research on precancerous lesions is far more limited (26–32). An overview of the most common bacteria found throughout the Correa cascade can be found in **Table 1**.

**Table 1:** Gastric dysbiosis throughout the Correa Cascade.

Most common bacteria	Status	References
Normal stomach		
*Prevotella, Streptococcus, Veillonella, Rothia, Haemophilus*	Abundant	(19–23)
Atrophic gastritis		
*Helicobacter pylori, Rothia, Peptostreptococcus, Abiotrophia, Streptococcus, Actinomyces, Granulicatella, Parvimonas, Capnocytophaga, Bacillus, Prevotella, Akkermansia, Gemella, Actinobacteria*	Enriched	(29, 31–34)
Gastric cancer		
*Helicobacter pylori, Akkermansia, Actinobacteria* Achromobacter, Clostridium, Citrobacter, Rhodococcus, Proteobacteria, Pseudomonadales*, Erysipelotrichales*, Neisseriales, *Streptococcus, Gemella, Nitrospirae*	Depleted Enriched	(26, 29, 32, 35 – 34, 36–39)

*H. Pylori negative cases.

### 2.1 *Helicobacter pylori*-triggered gastric carcinogenesis

One of the most clinically significant bacteria in the context of carcinogenesis is *H. pylori*, a mobile, microaerophilic, rod-shaped bacterium that is able to survive in the stomach environment (40). *H. pylori* its enclosed in a protective capsule, make it more resistant to adverse conditions and antibiotics, and so, its classified as a Gram-negative bacterium (41). Like other Gram-negative bacteria, *H. pylori* cell wall comprises of lipopolysaccharide (LPS), which has been linked to pathogenicity by contributing to stomach *H. pylori* colonization and persistence (42). LPS is composed of lipid A, a core oligosaccharide, and an O-specific chain (43). *H. pylori* LPS O-specific surface chain has a structural similarity to Lewis blood type antigens, which are commonly present in the human gastric mucosa (42–44). Molecular mimicry of Lewis antigens gives *H. pylori* the ability to evade host immune detection, allowing its survival and prevalence in the stomach (44). However, compared to other bacterial LPS, *H. pylori* LPS exhibits lower toxic activity (45).

*H. pylori* infects around 50% of global population and is associated with 79% of newly diagnosed cases of GC each year (46). Since there is enough evidence to conclude that it can cause cancer in humans, IARC categorizes *H. pylori* as a class I carcinogen (47). Different strains around the world are shown to have different effects on cancer risk (48). East Asia has high incidences of *H. pylori* infection and high incidences of GC, while some other highly infected populations in South Asia and Africa do not, possibly explained by genotype differences of *H. pylori* (48). Falush et al. (49) sequenced the strains from 27 geographical ethnic groups, and defined four major groups: hpEurope, hpEastAsia, hpAfrica1 and hpAfrica2, names associated with their geographic distribution. Later, hpEastAsia was subdivided into hspEAsia (East Asia), hspMaori (Polynesians) and hspAmerind (native Americans), while hpAfrica1 was divided into hspWAfrica (South Africa, Americas and West Africa) and hspSAfrica (South Africa) (49). From these clusters, population with hpEastAsia strain showed the highest rates of GC, in contrast with hpAfrica1 and hpAfrica2 (49). Virulent strains vary in the expression of cytotoxin-associated gene A (*CagA*) and vacuolating cytotoxin A (*VacA*), two of the most prominent virulence factors of *H. pylori* that contribute to inflammation, with these particularly aggressive strains (*VacA* and *CagA* positive [+]) linked to higher risk of GC and peptic ulcer disease (50–52) . East Asian type *CagA* is present in over 70% of patients with GC, and statistical studies have shown that East Asian type is more dangerous than *CagA* of the Western, explaining the high incidence of GC in East Asia (53, 54). In the hpAfrica2 strain, genomic structures are distinct, lacking the *CagA* gene, and hence explaining the high prevalence, but low rate, of GC (49, 55). On the other hand, the *V*ac*A* gene is present in all strains, varying in its vacuolating activity (56). S1/m1 and s1/m2 *VacA* strains have high or moderate toxin activity, a stronger inflammatory response, more DNA damage to epithelial cells, and higher carcinogenic potential (more associated with hspEAsia), while s2/m2 strains do not vacuolize (57). *H. pylori* adhesins, especially blood group antigen-binding adhesin gene A (*BabA*), play an important role in bacteria colonization of gastric epithelial cells and contribute to the development of GC (58).

**Figure 2:**
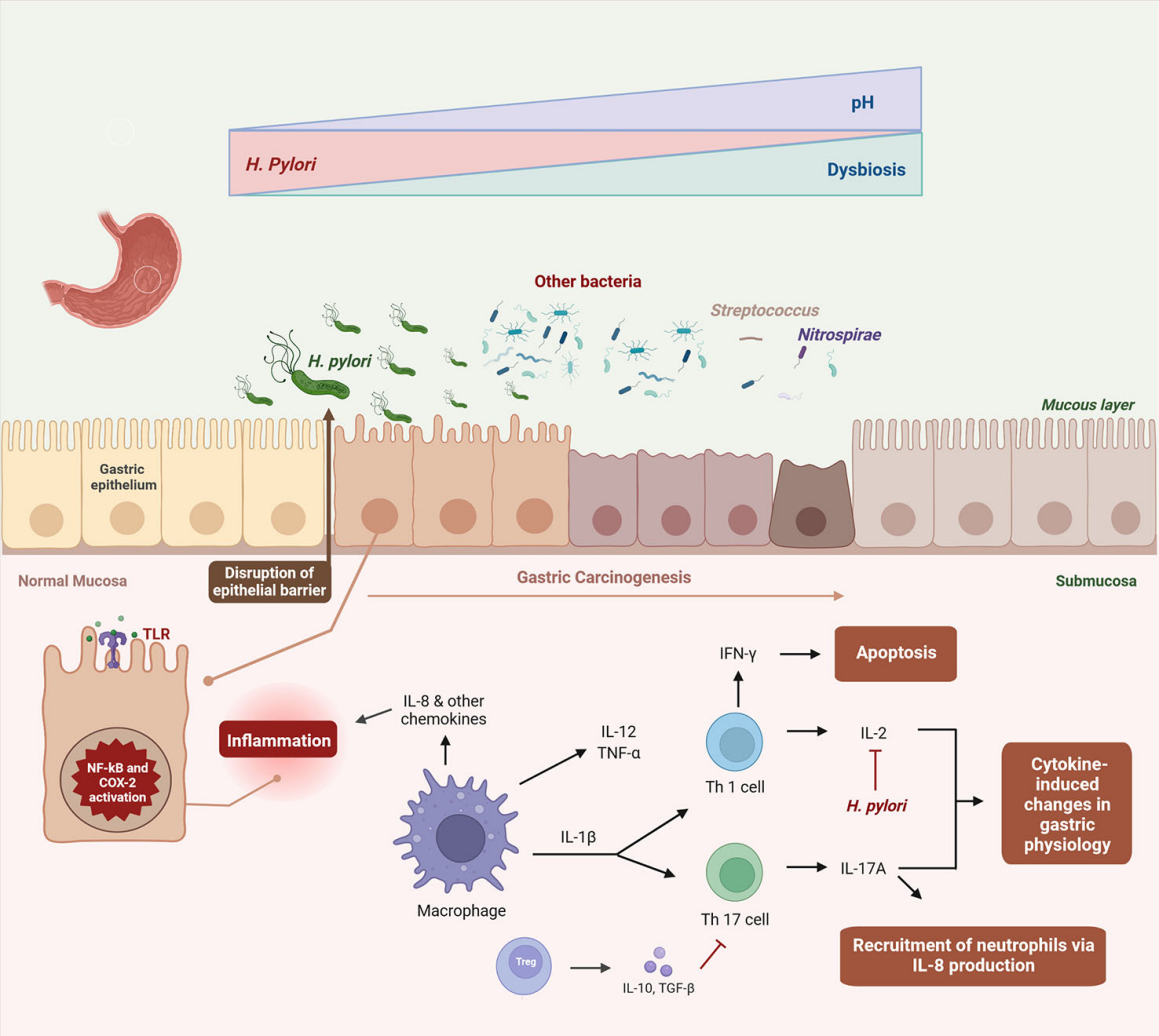
Dysbiosis-driven inflammation in gastric carcinogenesis. Once established in the gastric mucosa, *H. pylori* induces and maintains an inflammatory response, disrupting the epithelial barrier and altering the stomach acidity (27). In this altered environment, *H. pylori* colonization declines in the gastric carcinogenesis and other bacteria invade the gastric niche, culminating in dysbiosis (22, 32, 62–65). Virulence factors of *H. pylori* activate NF-κB and COX-2 pathways, promoting interleukin (IL)-8 synthesis and other immune cells, which are heavily recruited in response to infection, leading to cytokine-induced changes in gastric physiology (66–74). It also suppresses IL-2 synthesis, which is important for T-cell proliferation and survival (50, 75–77).

*H. pylori* produces the enzyme urease, which temporarily buffers the acidic environment by hydrolyzing urea into ammonia and carbon dioxide, forming a protection around the bacterium that allows it to survive the stomach acid (59). As mentioned above, *H. pylori* infection is considered the initial trigger for AG. Initiation of a chronic inflammatory process and direct toxic action of virulence factors are recognized as the key processes by which *H. pylori* leads to GC (60). However, the precise method by which this happens is still understood (61). Once established in the gastric mucosa, *H. pylori* induces inflammation, increasing the risk of gastric epithelium precancerous lesions (**Figure 2**) (27). After translocation, *CagA* activates NF-κB and promotes interleukin (IL)-8 synthesis, neutrophils are heavily recruited in response to infection and infiltrated into the stomach mucosa, which increases the severity of the inflammatory response (66). *VacA* protein destroys the tight junctions of epithelial cells, causing cell death by apoptosis and changes in cell signaling (50, 75–77). It also suppresses interleukin 2 (IL-2) synthesis, which is important for T-cell proliferation and survival, and decreased expression of surface IL-2 receptor (50, 75–77). Such outcomes are the result of *VacA* ability to suppress the activation of nuclear factor of activated T-cells (NFAT), a transcription factor that works as a central regulator of immune response genes and is crucial for efficient T-cell activation (78, 79). Ammonia produced by urea degradation causes damage at the cellular level and also induces the production and activation of inflammatory cytokines and phagocytes (60). Eradication of *H. pylori* may have a long-term positive effect on AG, as a meta-analysis indicated a regression of this condition after *H. pylori* treatment, although this effect was not detected for IM (80).

### 2.2 Non-*H. pylori* bacteria-mediated gastric carcinogenesis

A few studies found an association between certain gastric bacteria and precancerous lesions in the stomach (28–30). Due to the decreased production of gastric acid, various microorganisms are able to colonize the stomach in a patient with AG (35). Sung et al. (31) reported that *Rothia*, *Peptostreptococcus*, *Abiotrophia*, *Streptococcus*, *Actinomyces*, *Granulicatella* and *Parvimonas* were linked to AG and found in patients with IM after *H. pylori* eradication. Additionally, it has been postulated that the relative abundance of *Capnocytophaga*, *Bacillus*, and *Prevotella* bacteria rises as precancerous lesions evolve to dysplasia and GC, whereas the relative abundance of *Helicobacter* species declined (29). In a study by Ferreira et al. (26), *Achromobacter*, *Clostridium*, *Citrobacter* and *Rhodococcus* were found to be more common in Portuguese patients with GC than in those with chronic gastritis, in contrast with the notable decline of *H. pylori* in more advanced stages. Deng et al. (33) recently identified a substantial but non-statistically significant increase from 38% to 60% of *Proteobacteria* in biopsies from antral GC compared to biopsies from antrum gastritis, followed by a substantial reduction in *Actinobacteria*. In addition, they found an increase of *Pseudomonadales* and *Erysipelotrichales* in people with *H. pylori*-negative antral GC, compared to the infected individuals, who were enriched with *Neisseriales* (33). A recent study by Park et al. (34) reported a depletion of *Akkermansia* in GC stage, compared with his overabundance in the AG group, results consistent with other studies. This bacterium contributes to the maintenance of mucin and short-chain fatty acid balance by synthesizing a protein that degrades mucin, therefore promoting the barrier function (34). Hence, *Akkermansia* might play a preventative role in gastric carcinogenesis by impacting the integrity of the stomach mucosa (34). The authors also reported a significant increase of *Lactobacillus* and *Veillonella* in GC compared to other stages (34). *Lactobacillus* species are part of the human commensal gut bacteria (81). However, their overproliferation has been associated with IM or GC in humans and mice (26, 34). One possible explanation is that cancer cells are fueled by exogenous lactate supplied by lactic acid bacteria, which in turn promotes inflammation, angiogenesis, metastasis, and immune evasion (81).

In a study by Pimentel-Nunes et al. (32), *Streptococcus* and *Gemella* were present in patients with advanced AG, persist during the carcinogenesis cascade. Other studies support these findings, with *Streptococcus* being abundant in tumor tissue compared to normal (26, 35–38). *Nitrospirae* was also found in tumor tissue but absent in patients with chronic gastritis (39). Several *Nitrospirae* phylum members have been implicated in nitrate and nitrite metabolism (82). Consumption of nitrates is associated with an increased risk of developing GC, and it is possible that nitrosating bacteria, such as *Nitrospirae, Staphylococcus, Veillonella, Clostridium, Haemophilus, Streptococcus*, and *Neisseria*, can contribute to the formation of carcinogenic N-nitroso compounds (35, 83, 84).

### 2.3 Microbiota interplay in gastric carcinogenesis

The presence of *H. pylori* can alter the normal gastric microbiota, leading to dysbiosis and potentially the progression to GC (22, 62). Constant stimulation of the host immune system due to an imbalance in the gastric microbiota may lead to chronic inflammation of the gastric mucosa (62). It has been reported a decrease in the abundance of common phyla in the stomach when *H. pylori* is present (85). Animal studies have shown that the probiotic bacteria *Lactobacillus, Bifidobacterium*, and *Saccharomyces* have antagonistic effects against *H. pylori*, preventing from adhering, colonizing, and proliferating within the stomach, while *Eubacterium limosum* was shown to have the opposite effect (86–88). Three ways in which *H. pylori* interacts and disturbs the stomach microbiota have been proposed: 1) altering the stomach acidity, 2) providing substrates suitable for the proliferation of certain bacterial strains, and 3) modulating the host lifestyle and diet (89–92).

Overall, current research reports a greater diversity of gastric microbiota in healthy stomachs followed by a constant decrease throughout the cascade stages (63–65). The decline in mucosal microbial variety and richness may indicate that the disease is progressing (63–65) . *H. pylori* assumes its dominancy in initial atrophy stages, but not in IM (32).

Understanding how *H. pylori* and the microbiota affect the development of gastritis and cancer has come a long way. However, there have been discrepancies across studies, which could be related to the method used or differences in the host environment, such as diet, age, ethnicity, or genetics (93–95). In fact, some studies report considerable changes in stomach microbiota according to *H. pylori* status, while others fail to achieve statistical significance (96–98).

More detailed studies are still needed in well-defined human populations, to compare the topographic heterogeneity in microbial composition of the stomach of people with *H. pylori* infection with and without neoplastic lesions.

## 3 Dysbiosis-driven inflammation in gastric carcinogenesis: The role of NF-κB, TLR, and COX-2 pathways

In 2011, tumor-promoting inflammation was added to the hallmarks of cancer and the infiltration of tumors with both innate and adaptive immune system cells has been long recognized (99). Most major risk factors for common types of cancer, including chronic infections, tobacco, alcohol, or obesity, are triggers of chronic inflammation (100). Chronic inflammation driven by immune cells is a rather complex process requiring multiple cellular players (e.g., macrophages, lymphocytes) and pivotal regulators, including signaling pathways (e.g., NF-κB, JAK-STAT, TLR, MAPK), growth factors (e.g., VEGF, TGF-*ß*), as well as inflammatory factors (e.g., cytokines, chemokines), inflammasome, and inflammatory metabolites (e.g., prostaglandins, leukotrienes) , as reviewed by Zhao et al. (100). Tumor growth is promoted by an inflammatory response, which in turn is induced by inflammatory mediators that can attract immune cells to the lesion (101). That promotes the perfect conditions for the growth, invasion and metastization of tumor cells, known as the tumor microenvironment (101).

Emerging technologies such as single-cell RNA-sequencing (scRNA-seq) are delivering unprecedent insights of elusive immune-mediated mechanisms of gastric carcinogenesis by providing a comprehensive view of the cellular landscape and interactions within a tissue at a single-cell resolution, with the potential to contribute towards the development of more effective diagnostics and therapeutic targets (102–104).

In the stomach, the presence of inflammatory cells constitutes gastritis, which can evolve to atrophy and later to adenocarcinoma in a multistep process as aforementioned (105). This progression has been linked to gastric microbiota, since sterile mice colonized with three strains of Altered Schaedler’s flora (ASF) presented increased levels of pro-inflammatory mediators, as well as cancer-related genes, compared to aseptic mice (106).

In this section, the role of *H. pylori* in gastric inflammation will be explored, as well as the association between microbiota and major inflammatory players (NF-κB, TLRs, and COX-2).

### 3.1 *Helicobacter pylori*-triggered chronic gastritis

The main cause of chronic AG is the persistence of *H. pylori* infection (107). That requires a broad spectrum of adhesion factors, and *H. pylori* genomes from various strains contain over 30 genes encoding outer membrane proteins divided into Hop, *Helicobacter* outer membrane porins, and Hor, hop-related (108). *BabA*-mediated adherence of the bacterium, a member of the Hop family of proteins and the first adhesin discovered, is pH sensitive and allows the infection to adapt to shifts in stomach acidity resulting from chronic inflammation through mutations and other events in *BabA-*related genes (108, 109). Upon adhesion, the type IV secretion system (T4SS) components within the cagPAI pathogenicity island assemble a structure that mediates the transfer of *CagA* into the host cells in the epithelium, promoting the increased release of IL-1β and IL-8 (110, 111). The former allows a more effective inflammatory response, whereas the latter is a proinflammatory chemokine involved in neutrophils chemotaxis, quickly responding to stimulus from infectious agents and initializing the reaction (110). On the other hand, the same cagPAI strains that induce IL-1β also induce IL-10, an anti-inflammatory cytokine known to inhibit NF-κB activation and IL-8 expression, promoting the growth of bacteria (110). *H. pylori* response to neutrophils appears to vary from pro- to anti-inflammatory, depending on the integrity of T4SS and *cagPAI* (110). Additionally, loss of matrix metalloproteinase 7 (MMP-7), secreted mainly by epithelial cells, has been associated with significantly more severe gastric inflammation in mice infected with *H. pylori* (112). MMP-7 affects the extracellular matrix, potentially altering the microenvironment, and its loss leads to an increase in M1 macrophage activation and proinflammatory cytokines secretion by those cells (112).

Overall, the inflammatory microenvironment promoted by *H. pylori* can increase mutation rates, DNA damage through double strand breaks and aberrant DNA methylation, as well as other epigenetic changes (113). These alterations may lead to the progression of the gastric carcinogenesis cascade from chronic gastritis to atrophic gastritis, and residual DNA methylation after eradication may be involved in GC development (113). In its turn, *H. pylori* infection leads to the activation of anti-microbial mechanisms through the infiltration of macrophages and neutrophils into the gastric mucosa (114). These cells release the aforementioned chemokines and cytokines, reactive oxygen species (ROS) and reactive nitrogen species (RNS), increasing oxidative stress, MMPs, and PGs, such as PGE_2_, maintaining the tumor-promoting inflammatory microenvironment and enhancing genomic instability (114).

### 3.2 Microbiota-mediated NF-κB pathway in gastric carcinogenesis

The NF-κB family comprises five genes, *NF-κB1*, *NF-κB2*, *RelA*, *c-Rel*, and *RelB*, that encode seven proteins with a common Rel homology domain (RHD) able to mediate DNA binding, interaction with inhibitors and dimerization (115). These proteins remain predominantly in the cell cytoplasm due to their interaction with inhibitors, IκBs, which bind to the RHDs of NF-κB (115). A variety of stimulus can trigger NF-κB activation, including growth factors, cytokines, and signaling pathways, such as PI3K/Akt and Ras/MAPK (115). Upon activation, the NF-κB signaling pathway affects cellular proliferation and apoptosis, by targeting molecules such as Bcl2 and cyclins, as well as immune response and inflammation, by upregulating chemokines and cytokines as the ones mentioned in the previous section (116).

Induction of NF-κB pathway by *H. pylori* has been known for over 20 years (117). During infection, this pathway is readily employed to activate antibacterial immunity, playing an important role in microbiota-associated gastric tumorigenesis (116). In fact, in *H. pylori-*infected individuals, NF-κB activity was markedly increased compared to controls, suggesting an association between gastritis due to neutrophil infiltration and the activation of this pathway (118). On the other hand, NF-κB has been shown to be implicated in genomic instability through R-loop induction, associated with DNA damage and replication stress, and through DNA double strand breaks as a result of *H. pylori* infection mediated by T4SS (119, 120).

Countless molecules have been shown to interact with the NF-κB pathway in response to *H. pylori*, either by inhibiting it or enhancing its effects and playing a role in gastritis. For example, bacterial infection in the early development stage leads to microRNA (miR)-204 downregulation, resulting in decreased baculoviral IAP repeat-containing 2 (BIRC2) expression, a protein that promotes IκBα degradation (121). Consequently, this leads to NF-κB signaling enhancement by allowing its entrance in the nucleus (121). Similarly, loss of trefoil factor 1 (*TFF1*) expression, a tumor suppressor gene, leads to an increase of NF-κB transcription factors expression, modulating the inflammatory response, through the activation of the tumor necrosis factor (TNF) receptor 1 (TNFR1)/IκB kinase (IKK) pathway (122). Reconstitution of *TFF1* expression in cells led to a significantly suppression of the increase of NF-κB nuclear staining mediated by *H. pylori* (123). Accordingly, *Tff1*-knockout (KO) mice infected with the bacterium showed higher chronic inflammation compared to uninfected mice (123). In the same mouse model, increased levels of aurora kinase A (AURKA) were also associated with stronger chronic inflammation, resulting from the consequent increase of NF-κB phosphorylation and activation (124). Moreover, tumor necrosis factor receptor-associated factor-interacting protein with forkhead-associated domain (TIFA) was reported to bind to TRAF6 and TRAF2, forming TIFAsomes, which trigger NF-κB upon *H. pylori* infection of cells (125). On the other hand, tumor necrosis factor‐α‐induced protein‐8 like-2 (TIPE2) was identified as a negative regulator of this pathway, being able to maintain immune homeostasis in an independent manner (126). Furthermore, it has been shown to modulate HOX transcript antisense RNA (HOTAIR) levels, a long non-coding RNA enhanced in early stages of gastric tumorigenesis, particularly gastritis (126).

In contrast, there are also numerous molecules influenced by the NF-κB pathway upon bacterial infection, mainly with increased expressions, namely the oncogenes β-catenin and c-Myc (127), *RASAL2* (128), Rev-erbα (127), dopamine- and cAMP-regulated phosphoprotein Mr 32 kDa (DARPP-32), whose overexpression occurs early in IM (129), miR233-3p, which decreases AT-rich interactive domain-containing protein A1 (ARID1A) expression and promoting proliferation and migration of cells directly associated with CagA (130). This *H. pylori* virulent factor induces the NF-κB-mediation of let-7a downregulation, resulting in increased human telomerase reverse transcriptase (hTERT) expression in AG and IM, indicating a role of this marker in cancer initiation (131).

Regarding other bacterial species, bacterial abundance and colonization rates were increased in *Nfkb2*-KO mice compared to control mice infected with *Helicobacter felis (H. felis)* (132). In fact, NF- κB2 signaling disruption appeared to be crucial for gastric atrophy development in response to that infection (132). In another study by Shibata et al. (133), a mice model with IKK depletion in gastric cells presented faster *H. felis*-dependent progression to dysplasia, which was associated with increased apoptosis and necrosis due to cellular stress induced by the bacterium. The latter process involved upregulation of IL-1 and C-X-C motif ligand 2 (CXCL2), a chemokine involved in leukocytes chemotaxis, consequently enhancing chronic inflammation (133). Furthermore, as a probiotic, *Lactobacillus casei* was shown to inhibit cell proliferation and promote apoptosis through NF-κB signaling modulation (134).

### 3.3 Microbial activation of toll-like receptors

TLRs constitute a group of type I transmembrane glycosylated proteins able to detect microbes or products from microorganisms either outside (TLR1, TLR2, TLR4, TLR5, and TLR10) or inside the cell (TLR3, TLR7, TLR8, TLR9) (135). These proteins are able to sense pathogen-associated molecular patterns (PAMPs) or microbe-associated molecular patterns (MAMPs), leading to the activation of downstream signaling pathways in response to bacteria, fungi, protozoa or viruses (135, 136). TLR signaling can be either MyD88- or Tikk/IL-1 receptor (TIR) domain-containing adaptor inducing interferon-β (TRIF)-dependent, ultimately reaching the same endpoint: NF-κB activation and production of type I interferons, chemokines, and cytokines to fight infection (135). TLR expression, particularly TLR2, TLR4, and TLR5 has been found upregulated throughout gastric carcinogenesis cascade, including IM, dysplasia, and adenocarcinoma, showing an association between these receptors and the development of lesions in the stomach (137–139).

The relationship between TLRs and *H. pylori* has been extensively explored and reviewed throughout the years (140–144). After colonization, bacteria interact with TLR1, TLR2, TLR4, TLR5, TLR6, and TLR10 in the cell surface, as well as TLR9 in the intracellular environment, whose expression is dynamically regulated (145, 146). In fact, *H, pylori* infection has been associated with increased expression of some of these transmembrane receptors (147, 148). Protein CagL, one of T4SS components, acts as a TLR5 activator, independent of flagellin (149). On the other hand, TLR2 and TLR4 are ligands for *H. pylori* LPS, the former resulting in overexpression of IL-8 and the latter leading to the release of IL-12 and IL-1β, which induce differentiation of T helper (Th) 1 and Th17 cells, producers of pro-inflammatory cytokines, IL-2, IL-6, and IL-18, an IL-1β antagonist, thus contributing to immune response evasion (150, 151). IFN-γ is primarily produced by Th1 cells (152). Osaki et al. (153) conducted experiments on mice and human gastric cells and found that this cytokine induced apoptosis, or programmed cell death, in gastric epithelial cells. Using a mouse model of *H. pylori* infection, the authors found that mice lacking IFN-γ had reduced inflammation and less metaplasia compared to wild-type mice (153). Moreover, its expression was increased in patients with IM (153). Therefore, IFN-γ seems to be required for the progression of IM and its induction of gastric epithelial cell death appears to contribute to this process (153). Atrophy and atrophic gastritis can result from increased expression of IL-1β, which decreases the activity of the gastric proton pump, and release of IL-17A by Th17 cells, inducing parietal cell apoptosis (154–156). Regulatory T cells, also known as Tregs, play a crucial role in maintaining immune homeostasis and preventing excessive immune responses to gastric microbiota, being able to suppress the activation and proliferation of Th7 cells (157–159). However, imbalances in gastric microbiota can directly impact Treg cell number and function, contributing to the perpetuation of inflammation (160). Patients with gastritis and active *H. pylori* exhibit significantly elevated expression of Tregs, TGF-β, and IL-10 compared to non-infected individuals (161, 162). Therefore, the presence of these cells in the mucosa may inhibit the immune response, resulting in the prolonged presence of infection (163). The exact mechanisms by which Treg cells regulate immune responses and the interplay between these cells, gastric microbiota, and gastric inflammation is still not fully understood and is an active area of research.

Evidence suggests that using TLR4 antagonists, such as the TAK-242 molecule, may contribute to the treatment of *H. pylori*-associated gastric lesions, since it binds to the receptor and disrupts its interaction with LPS, inhibiting the activation of downstream signaling pathways (164). Concerning TLR9, its expression has been found increased in response to *H.* pylori DNA, potentiating cellular proliferation, migration and invasion (165). Moreover, in gastric atrophy, intracellular TLR9 is activated due to the release of damage-associated molecular patterns (DAMPs) from cellular and bacterial debris (166).

Numerous studies have focused on the impact and role of TLR polymorphisms in GC and precancerous lesions (167, 168). SNPs in TLR-encoding genes, namely *TLR2, TLR4, TLR5*, *TLR9*, and *TLR10* have been associated with *H. pylori* infection risk and/or chronic gastritis (166, 169–173). On the other hand, carriers of the *TLR1* rs4833095 T allele presented decreased risk of infection and chronic atrophic gastritis (173, 174). Lower risk of the latter was also observed in individuals carrying the *TLR10* rs10004195 T allele, suggesting a potential role of these SNPs in gastric pathogenesis associated with *H. pylori* (174, 175).

Regarding non-*H. pylori* bacteria, *Neisseria subflava* has been shown to interact with TLR4 through LPS, resulting in IL-8 expression, whereas *TLR9*-KO mice were protected from gastric inflammation and hyperplasia induced by *H. felis* (176, 177).

### 3.4 Cyclooxygenase-2 role in gastritis-associated inflammation

COX-2 is an enzyme with dual enzymatic activity, cyclooxygenase and peroxidase, responsible for catalyzing the rate-limiting step in prostaglandins synthesis: the addition of molecular oxygen into arachidonic acid (178). Prostaglandins are biologically active lipids involved in a variety of physiological processes, including inflammation, and PGE_2_ is the best known and most abundant, appearing to affect virtually all hallmarks of cancer (178). COX-2 is an enzyme with dual enzymatic activity, cyclooxygenase and peroxidase, responsible for catalyzing the rate-limiting step in prostaglandins synthesis: the addition of molecular oxygen into arachidonic acid (178). Prostaglandins are biologically active lipids involved in a variety of physiological processes, including inflammation, and PGE_2_ is the best known and most abundant, appearing to affect virtually all hallmarks of cancer (178).

The first description of COX-2 expression in GC goes back to 1997 and many studies reported an increase of its levels in *H. pylori*-mediated gastritis, suggesting that high COX-2 levels is an early event induced by this bacteria in gastric carcinogenesis (67–74). Nevertheless, the publication period of those studies comprised mainly the early 2000’s, whereas most recent reports have been focusing on cancer prevention through COX-2 inhibition and drug repurposing (179). A review on the latter topic is available in the literature (180). In gastric adenocarcinomas, high levels of COX-2 expression are linked to lymph node metastasis and depth of invasion, suggesting that prostanoids generated by this enzyme increase the aggressive behavior of these tumors (16). COX-2 expression is particularly high in GC, and it is also higher in dysplastic lesions compared with normal mucosal tissue, revealing the contribution of COX-2 in gastric carcinogenesis even at the preinvasive state (16). Concerning its association with microbiota, full induction of this enzyme has been demonstrated to be dependent on direct bacterial contact, a functional T4SS system, and epidermal growth factor receptor (EGFR) activation (181). On the other hand, PGE_2_ signaling, and *H. pylori* infection are required for the induction of the chemokine C-C motif ligand 2 (CCL2), responsible for attracting macrophages to the site (181).

Dysregulation of the COX-2 pathway has been reported in GC, and genetic variants of these genes have been identified as susceptibility biomarkers for the development of this malignancy, as well as gastritis and IM (182–185). Consequently, higher PGE_2_ levels have been reported in the gastric juice of patients with chronic AG and IM compared to controls (186). Furthermore, treatment of advanced gastric lesions with a selective COX-2 inhibitor alone, celecoxib, revealed beneficial effects on the regression of the disease, like what was seen with *H. pylori* eradication (187). There is evidence on the interplay between TLR/MyD88 signaling and COX-2/PGE_2_ pathway, reviewed in (188), suggesting the former promotes tumorigenesis by activating the latter and generating an inflammatory microenvironment. Additionally, NF-κB has also been shown to mediate COX-2 expression, resulting in higher PGE_2_ levels (189, 190). Parallelly, the latter pathway has been linked to IL-8 production in gastric epithelial cells (191).

## 4 Liquid biopsies as a promising source of dysbiosis-derived biomarkers in early gastric cancer development

Liquid biopsies have revolutionized the field of Precision Oncology, easing the collection of samples and biomarker assessment through minimally invasive procedures and allowing continuous monitoring of a tumor over time with increased sensibility (192). Overall, the most frequently used in clinical practice are the serum markers carcinoembryonic antigen (CEA), a glycoprotein, and carbohydrate antigen (CA)19-9, both found overexpressed in GC (193). However, these proteins, as well as other common markers such as alpha-fetoprotein, CA125 and CA72-4, lack sensitivity and sensibility, with the combined detection of CEA, CA19-9 and CA72-4 presenting greater diagnostic value (194–196). The most assessed biomarkers targeting earlier stages of gastric carcinogenesis, namely AG include serum pepsinogen (PG) levels, gastrin, and anti-*H. pylori* antibody detection (197). The performance of the combination of these three biomarkers as a test for AG diagnosis has been reported in a meta-analysis by Zagari et al. (197), proving to be a reliable tool, but still requiring cost-effectiveness analysis. Other markers include neopterin and C-reactive protein (CRP), early inflammation molecules that increase significantly in IM and atrophy compared with chronic gastritis alone (198).

Choi et al. (199), using a metagenomic approach to characterize gastric juice samples, identified *Helicobacter* and *Streptococcus* as the most prevalent bacterial genera in gastric malignancies or ulcers when compared to controls. Furthermore, *H. pylori*-derived extracellular vesicles (EVs) were abundant and shown to contain *CagA* and *VacA*, inducing the production of cytokines, such as TNF-α, IL-6, IL-1β, and IL-8, and promoting inflammation (199). More recently, the gastric mucosa and gastric juice microbial composition were proven to change considerably from superficial gastritis to GC, with a panel including *Gemella, Haemophilus, Peptostreptococcus, Streptococcus*, and *Veillonella*, validated in an independent set with robust performance (200). Interestingly, the bacterial community profile in the gastric mucosa converged to the one found in gastric juice throughout disease progression, suggesting an interchange of microbes between the tissue and the liquid biopsy in direct contact (200). Thus, gastric fluid appears as a promising source of biomarkers, since it reflects the functional state of the stomach, potentially complementing and enhancing the accuracy of endoscopy as the gold standard for precancerous stomach and GC detection and surveillance (11). In fact, a novel device that allows *H. pylori* detection and corpus AG diagnosis during endoscopy from gastric juice, regardless of proton inhibitor therapy or previous eradication, has been presented, positively impacting the use of health resources and patients comfort (201–203). Furthermore, a systematic review was recently published on the role of non-blood liquid biopsies in GC development and detection (11).

## 5 Clinical utility of dysbiosis-derived biomarkers in gastric cancer-targeted screening and personalized surveillance

Although several advanced techniques have been developed aiming to enhance the accuracy of AG diagnosis by improving the visualization of gastric pre-malignant changes, including autofluorescence imaging (AFI), chromoendoscopy, high-definition endoscopy with magnification, and narrow band imaging (NBI), studies exploring the clinical utility of dysbiosis-driven inflammatory biomarkers have been rather scarce (204).

Overall, *H. pylori* role in inflammation and gastric carcinogenesis has been extensively explored, unlike other gastric bacteria. Nevertheless, a study by Liu et al. (17) reported other relevant bacteria with robust performance to distinguish superficial gastritis from AG (area under the ROC curve [AUC] = 0.86), IM (AUC = 0.71), and GC (AUC = 0.85), despite also including *Helicobacter* (14). Furthermore, the combination of six bacterial genera from tongue coating, excluding *Helicobacter*, was able to distinguish GC patients from healthy controls with a median area under the curve (AUC) value of 0.88, proving the potential of non-*H. pylori* bacteria as powerful diagnostic biomarkers for the detection of gastric pathologies (205).

## 6 Conclusion

Over a decade ago tumor-promoting inflammation was defined as an enabling characteristic fundamentally triggering the activation of the eight hallmark capabilities necessary for tumor growth and progression. In this review, we highlighted the role of inflammation in bridging the gap between gastric dysbiosis and precancerous stomach with chronic infection with, but not limited to, *H. pylori* eliciting a strong proinflammatory Th1 cell response in early stages of gastric carcinogenesis with several inflammatory-related biomarkers identified, namely NF-κB, TNF-α, IL-1β and IL-6. Considering most studies offered a mechanistic perspective, the clinical utility of the microbial-triggered inflammatory biomarkers in precancerous stomach tailored detection and management should be warranted in future studies. Furthermore, promising data recently published, albeit sparse, support not only the diagnostic value of microbial biomarkers but also the potential of gastric juice as a liquid biopsy. If validated in future studies, the non-invasive detection of inflammation-related biomarkers can push forward the field towards the concept of Functional Endoscopy and personalization of care in GC early diagnosis and surveillance.

## Author contributions

CP, MD-R, and PP-N contributed to the conception and design of this review article. CL and TA prepared the first draft of the manuscript. All authors contributed to the article and approved the submitted version.
